# Revealing the Defect-Dominated Electron Scattering in Mg_3_Sb_2_-Based Thermoelectric Materials

**DOI:** 10.34133/2022/9875329

**Published:** 2022-10-14

**Authors:** Jucai Jia, Yan Zhou, Xiaoxi Chen, Wenhua Xue, Hulei Yu, Jing Li, Shizhen Zhi, Chen Chen, Jian Wang, Shuaihang Hou, Xingjun Liu, Yumei Wang, Feng Cao, Yue Chen, Jun Mao, Qian Zhang

**Affiliations:** ^1^School of Materials Science and Engineering, and Institute of Materials Genome & Big Data, Harbin Institute of Technology (Shenzhen), Shenzhen 518055, China; ^2^Center for Device Thermography and Reliability (CDTR), H. H. Wills Physics Laboratory, University of Bristol, Tyndall Avenue, Bristol BS8 1TL, UK; ^3^Institute of Nuclear Physics and Chemistry, China Academy of Engineering Physics, Mianyang 621900, China; ^4^Beijing National Laboratory for Condensed Matter Physics, Institute of Physics, Chinese Academy of Sciences, 100190 Beijing, China; ^5^Department of Mechanical Engineering, The University of Hong Kong, Pokfulam Road, Hong Kong SAR, China; ^6^School of Science, Harbin Institute of Technology (Shenzhen), Shenzhen 518055, China; ^7^State Key Laboratory of Advanced Welding and Joining, Harbin Institute of Technology, Harbin 150001, China

## Abstract

The thermoelectric parameters are essentially governed by electron and phonon transport. Since the carrier scattering mechanism plays a decisive role in electron transport, it is of great significance for the electrical properties of thermoelectric materials. As a typical example, the defect-dominated carrier scattering mechanism can significantly impact the room-temperature electron mobility of n-type Mg_3_Sb_2_-based materials. However, the origin of such a defect scattering mechanism is still controversial. Herein, the existence of the Mg vacancies and Mg interstitials has been identified by synchrotron powder X-ray diffraction. The relationship among the point defects, chemical compositions, and synthesis conditions in Mg_3_Sb_2_-based materials has been revealed. By further introducing the point defects without affecting the grain size via neutron irradiation, the thermally activated electrical conductivity can be reproduced. Our results demonstrate that the point defects scattering of electrons is important in the n-type Mg_3_Sb_2_-based materials.

## 1. Introduction

Thermoelectric materials can realize the direct conversion between thermal energy and electricity and vice versa. Solid-state thermoelectric modules have been applied for power generation and electronic refrigeration [1–4]. Thermoelectric performance of a single material is evaluated by the figure of merit *zT* (*zT* = *S*^2^*σT*/*κ*), where *S* is the Seebeck coefficient, *σ* is the electrical conductivity, *κ* is the thermal conductivity, and *T* is the absolute temperature [5, 6]. Essentially, the thermoelectric parameters (*S*, *σ*, and *κ*) are governed by the transport of electrons and phonons. In polycrystalline materials, the existence of crystal defects, e.g., grain boundaries, dislocations, and point defects, is usually unavoidable. Such defects will distort the perfect crystal structure and scatter electrons and phonons [7]. In other words, the defect-dominated (electron and phonon) scattering mechanism will play a pivotal role in the thermoelectric transport properties. It is well known that phonon scattering by defects is quite notable and can substantially reduce the lattice thermal conductivity [8–11]. As a result, phonon engineering by introducing defects has been widely adopted to improve the *zT* of thermoelectric materials [12–18].

Similarly, the defects can significantly impact electron transport in thermoelectric materials. Usually, grain boundary scattering [19, 20], ionized impurity scattering (by charged point defects and ionized impurities) [21], and alloying scattering (by neutral substitutional point defects) [22, 23] have been regarded as the important defect-dominated scattering mechanisms in thermoelectric materials. However, thermoelectric materials are often synthesized in the thermodynamical nonequilibrium methods (e.g., quenching, arc-melting, and mechanical alloying [24]), which unavoidably lead to the coexistence of a high concentration of various defects. In this scenario, it is extremely difficult to distinguish how a specific type of defect scatters the electrons.

N-type Mg_3_Sb_2_-based materials exhibit outstanding thermoelectric performance [25–34]. Remarkable device performance for power generation and cooling has been demonstrated [33, 35–40]. However, it has been found that the room-temperature electron mobility of Mg_3_Sb_2_ is highly sensitive to the preparation conditions and chemical compositions [19, 41–46]. Such a unique phenomenon has been ascribed to the defect-dominated electron transport, but the underlying electron-scattering mechanism is still controversial. Recently, grain boundary scattering has been regarded as the dominant electron scattering mechanism [19, 20, 44, 47]. With the enlarged grain sizes, by increasing the sintering temperature, a noticeable enhancement in electron mobility can be realized [19, 44]. In addition, the abnormal temperature dependence of electron mobility is eliminated in the single crystals that further support this viewpoint [30, 45, 46]. It should be pointed out that the ionized impurity scattering due to the Mg vacancies is also proposed as an important electron scattering mechanism [41, 43]. By doping with a very low concentration of the transition metal elements, the room-temperature electron mobility can be effectively improved [25, 28, 41, 43, 48].

In terms of defect characterizations, information regarding the grain size can be easily obtained by optical and electron microscopy. On the contrary, characterizations of point defects are much more challenging [49, 50]. Up to now, only a few experimental studies related to point defects in Mg_3_Sb_2_-based materials have been reported [51, 52]. In addition, it is well known that different crystal defects are both sensitive to the preparation temperatures and chemical compositions. Therefore, increasing the sintering temperature or preparing the single crystal at equilibrium condition not only reduces or eliminates the grain boundaries but also unavoidably reduces the concentration of other defects. In other words, due to the difficulty of tuning the defects independently, identifying the defect-dominated electron scattering mechanism is quite challenging.

Herein, we revisit the issue of the electron scattering mechanism in n-type Mg_3_Sb_2_-based materials. By carefully characterizing the microstructures and point defects of the n-type Mg_3_Sb_2_-based samples, our results show that the concentrations of Mg vacancies and Mg interstitials are sensitive to the preparation conditions and chemical compositions. In addition, by intentionally introducing the point defects without affecting the grain size via neutron irradiation, we can reproduce the thermal activation of electrical conductivity. Our results demonstrate that point defects play an appreciable role in the electrical properties of Mg_3_Sb_2_-based materials.

## 2. Result and Discussion

### 2.1. Abnormal Electrical Properties around Room Temperature

**Figure 1:**
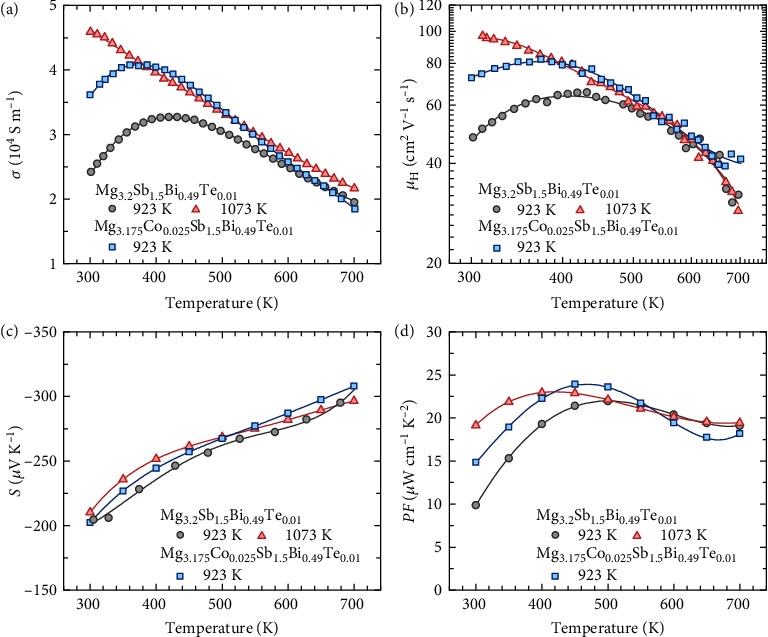
Electrical transport properties of n-type Mg_3_Sb_2_-based compounds. (a) Electrical conductivity, (b) hall mobility, (c) the Seebeck coefficient, (d) power factor of Mg_3.2_Sb_1.5_Bi_0.49_Te_0.01_ that hot-pressed at 923 K and 1073 K, and Mg_3.175_Co_0.025_Sb_1.5_Bi_0.49_Te_0.01_ that hot-pressed at 923 K.

Two samples of Mg_3.2_Sb_1.5_Bi_0.49_Te_0.01_ are hot-pressed at 923 and 1073 K, respectively. Another sample of Mg_3.175_Co_0.025_Sb_1.5_Bi_0.49_Te_0.01_ is hot-pressed at 923 K. The electrical properties of the three samples are shown in Figure 1. Distinct differences in the temperature dependence of electrical conductivity near room temperature can be observed (Figure 1(a)). Mg_3.2_Sb_1.5_Bi_0.49_Te_0.01_ that is prepared at 923 K shows thermally activated electrical conductivity below 500 K, inconsistent with the acoustic phonon scattering mechanism, resulting in a lower room-temperature electrical conductivity. In contrast, the room-temperature electrical conductivities of Mg_3.175_Co_0.025_Sb_1.5_Bi_0.49_Te_0.01_ and Mg_3.2_Sb_1.5_Bi_0.49_Te_0.01_ (prepared at 1073 K) are much higher. According to the Hall measurement, the room-temperature electron concentrations of the three samples are comparable (Figure S1, Supporting Information). Therefore, the disparity in the temperature dependence of electrical conductivity mainly originates from the difference in electron mobilities, as shown in Figure 1(b). The room-temperature electron mobility is as high as ~78 cm^2^ V^−1^ s^−1^ for Mg_3.175_Co_0.025_Sb_1.5_Bi_0.49_Te_0.01_ while it is only ~48 cm^2^ V^−1^ s^−1^ for Mg_3.2_Sb_1.5_Bi_0.49_Te_0.01_ (prepared at 923 K). A higher room-temperature electron mobility of ~97 cm^2^ V^−1^ s^−1^ is obtained for Mg_3.2_Sb_1.5_Bi_0.49_Te_0.01_ (prepared at 1073 K). In principle, the temperature dependence of electron mobility is mainly determined by the electron scattering mechanism. The distinct discrepancy in the temperature dependences of electron mobility reveals the different electron scattering mechanisms. In addition, a similar Seebeck coefficient is observed for all the samples (Figure 1(c)). As a result, noticeably enhanced room-temperature power factors have been achieved for Mg_3.175_Co_0.025_Sb_1.5_Bi_0.49_Te_0.01_ (~14.8 *μ*W cm^−1^ K^−2^) and Mg_3.2_Sb_1.5_Bi_0.49_Te_0.01_ prepared at 1073 K (~19.1 *μ*W cm^−1^ K^−2^) compared to that of Mg_3.2_Sb_1.5_Bi_0.49_Te_0.01_ prepared at 923 K (~9.8 *μ*W cm^−1^ K^−2^), as shown Figure 1(d). The results obtained in this work are in good agreement with the previous reports [19, 42–44]. In other words, doping Co at the Mg site and increasing the preparation temperature are both effective in improving the room-temperature electrical properties of n-type Mg_3_Sb_2_-based materials.

### 2.2. Grain Size and Elemental Distribution

**Figure 2:**
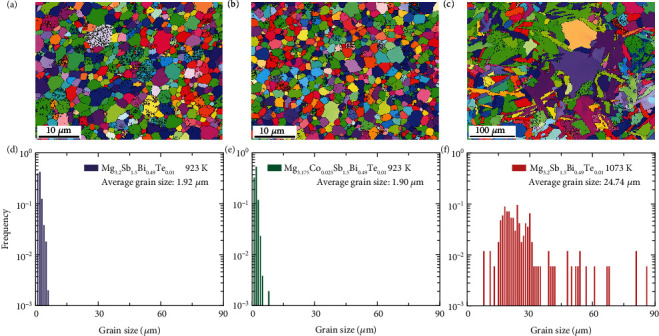
Grain size distribution. (a–c) EBSD crystal-orientation maps and (d–f) frequency statistics of grains size for Mg_3.2_Sb_1.5_Bi_0.49_Te_0.01_ that hot-pressed at 923 K and 1073 K, and Mg_3.175_Co_0.025_Sb_1.5_Bi_0.49_Te_0.01_ that hot-pressed at 923 K.

Since grain boundary scattering has been proposed as the dominant electron scattering mechanism in the Mg_3_Sb_2_-based materials, quantifying the variations in the grain size of these samples is necessary. Therefore, the electron backscatter diffraction (EBSD) characterization has been performed, and the results are shown in Figure 2 and Figure S2 (Supporting Information). Mg_3.2_Sb_1.5_Bi_0.49_Te_0.01_ (prepared at 923 K) has an average grain size of ~1.9 *μ*m (Figures 2(a) and 2(d)). In comparison, Mg_3.2_Sb_1.5_Bi_0.49_Te_0.01_ (prepared at 1073 K) has an average grain size of ~24.7 *μ*m (Figures 2(c) and 2(f)). In other words, there is a substantial grain size enhancement when the preparation temperature increases from 923 to 1073 K, and it is in good agreement with the previous reports [19, 44]. However, Mg_3.175_Co_0.025_Sb_1.5_Bi_0.49_Te_0.01_ has an average grain size of ~1.9 *μ*m, which is similar to that of the Mg_3.2_Sb_1.5_Bi_0.49_Te_0.01_ (prepared at 923 K), as shown in Figures 2(b) and 2(e). Therefore, Co-doping at the Mg site does not change the average grain size, and this is different from the report of Nb-doped Mg_3_Sb_2_ [53]. This result is reasonable considering that the doping concentration of Co is relatively low (~0.8 at.%), and the hot-pressing temperature is identical to the prepared Mg_3.2_Sb_1.5_Bi_0.49_Te_0.01_.

**Figure 3:**
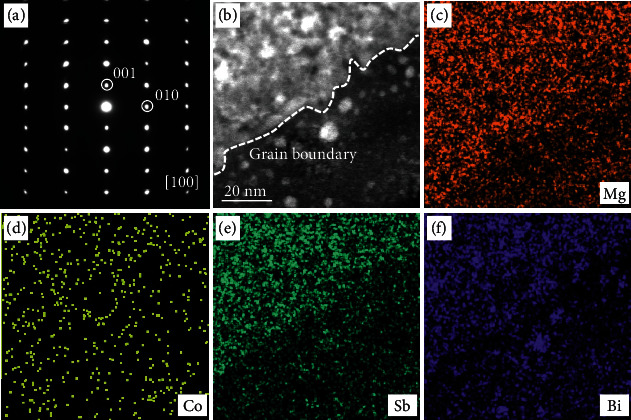
Elemental distribution around the grain boundary. (a) SAED pattern index as [100] direction, (b) HAADF-STEM image, and (c-f) EDS mapping images of the Mg_3.175_Co_0.025_Sb_1.5_Bi_0.49_Te_0.01._

It was speculated that the transition metal element was preferentially segregated at the grain boundary [19]. In this scenario, the potential barrier of the grain boundary will be reduced, which can alleviate the grain boundary scattering and improve electron mobility [20]. To verify this assumption, detailed elemental distribution near the grain boundary region in the Co-doped Mg_3.2_Sb_1.5_Bi_0.5_ is further characterized using transmission electron microscopy (TEM).  Figure 3(a) displays a selected area electron diffraction (SAED) pattern of the Co-doped Mg_3_Sb_1.5_Bi_0.5_, and it can be indexed as [100] direction with a hexagonal structure of the $P\bar{3}m1$. A clear grain boundary is identified, as shown in Figure 3(b). The elemental composition mapping by energy dispersive spectroscopy (EDS) has been conducted, and the results are shown in Figure 3(c)–3(f). As can be seen, the Co atoms distribute uniformly in the sample without preferential segregation at the grain boundary (Figure 3(d)). It should be pointed out that the white nanoparticles in Figure 3(b) are Bi-rich (Figure 3(f)) instead of Co-rich. In addition, energy dispersive spectroscopy mapping inside the grain also shows similar results, i.e., the distribution of the Co atoms within the grain is uniform (Figure S3, Supporting Information). Our results are different from the report of Nb-doped Mg_3_Sb_2_ [53], where Nb impurity phases mainly segregate at the grain boundary. The discrepancy can be partially attributed to the notable difference in the doping concentration, i.e., the doping concentration of Nb is as high as ~3.33 at.% while that of Co is only ~0.8 at.%. The solubility of the transition metal elements in the Mg_3_Sb_2_-based materials is low, so a high doping concentration results in the formation of the impurity phases. In this case, the transition metal impurity phase will be trapped at the grain boundary. However, it should be pointed out that even when the doping concentration of Co is rather low and Co does not segregate at the grain boundary, its effect on the temperature dependence of electron mobility is still very significant.

### 2.3. Point Defect Characterizations

The point defect plays an essential role in the electrical properties of Mg_3_Sb_2_-based materials. The Mg vacancies have a low defect formation energy and are the “killer” defects that limit the n-type doping [25, 54–56]. It explains why tuning the stoichiometry, i.e., controlling the concentration of Mg vacancies, is critical for determining the conduction type (n-type or p-type) of the Mg_3_Sb_2_-based materials [25, 57, 58]. In addition to the Mg vacancies, the Mg interstitials [25] and also the defect complex (e.g., Frenkel defect) [51, 59] in the Mg_3_Sb_2_-based materials have also been reported. However, it is noted that there are controversies on the existence of the Mg interstitials and defect complex [52, 60]. Therefore, experimentally identifying the point defects in the n-type Mg_3_Sb_2_-based materials is necessary.

**Figure 4:**
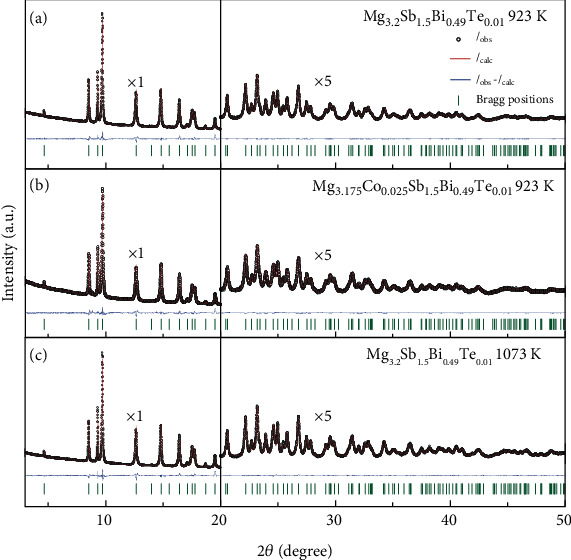
Rietveld refinement of the synchrotron powder X-ray diffraction patterns. (a) Mg_3.2_Sb_1.5_Bi_0.49_Te_0.01_ that hot-pressed at 923 K, (b) Mg_3.175_Co_0.025_Sb_1.5_Bi_0.49_Te_0.01_ that hot-pressed at 923 K, and (c) Mg_3.2_Sb_1.5_Bi_0.49_Te_0.01_ that hot-pressed at 1073 K. The data after 20 degrees is magnified five times to show details more clearly.

In our study, the synchrotron powder X-ray diffraction (SPXRD) measurements are conducted, and the Rietveld refinements are shown in Figure 4. Details for the atomic sites and the occupancy of Mg atoms at the Mg (1) site, Mg(2) site, and interstitial site Mg_I_ are shown in Table 1. It can be seen that there are appreciable differences in the Mg atom occupancy among the three samples. For Mg_3.2_Sb_1.5_Bi_0.49_Te_0.01_ which is prepared at 923 K, the occupancy of the Mg(1) site is only ~0.921, and the interstitial site Mg_I_ exhibits an occupancy of 0.058. It demonstrates the existence of the Mg vacancies and Mg interstitials. In comparison, the occupancy of the Mg(1) site increases to ~0.935, and that of the interstitial site Mg_I_ decreases to 0.039 in Mg_3.175_Co_0.025_Sb_1.5_Bi_0.49_Te_0.01_ that is prepared at 923 K. It means that Co-doping can effectively reduce the Mg vacancies and Mg interstitials in Mg_3_Sb_2_-based materials. For Mg_3.2_Sb_1.5_Bi_0.49_Te_0.01_ that is prepared at 1073 K, the occupancy of the Mg(1) site is as high as ~0.979, and the interstitial site Mg_I_ exhibits an occupancy of 0.047. In other words, the higher preparation temperature can effectively reduce the Mg vacancies and the Mg interstitials in the Mg_3_Sb_2_-based materials. Usually, the point defects concentration in thermodynamical equilibrium increases with the temperatures. In this work, the Mg_3_Sb_2_-based materials are prepared by mechanical alloying and hot pressing. Due to the high mechanical energy during ball-milling, a high concentration of oversaturated point defects can be produced [61]. These point defects will be suppressed after notable atomic diffusion during hot pressing [62]. In this case, it can explain why a higher preparation temperature will result in a lower concentration of Mg vacancies. Combining the results of electrical transport measurements and point defect characterizations, Mg_3.175_Co_0.025_Sb_1.5_Bi_0.49_Te_0.01_ and Mg_3.2_Sb_1.5_Bi_0.49_Te_0.01_ (prepared at 1073 K) with reduced concentration of point defects exhibit higher electron mobilities.

It is noted that the single-crystalline Mg_3_Sb_2_ (prepared in the Mg-rich condition) also exhibits a very high occupancy of 0.993 at the Mg (1) site, indicating the concentration of Mg vacancy is negligible [51]. In other words, the n-type single-crystalline Mg_3_Sb_2-*x*_Bi*_x_* is not only free of grain boundary but is also nearly free of Mg vacancies. This can also explain why the room-temperature electrical conductivity of single crystals is dominated by acoustic phonon scattering [32, 47, 48]. In addition, the single-crystalline Mg_3_Sb_2_ that is prepared in the Mg-poor condition shows p-type conduction [63], indicating the existence of Mg vacancies. Later, characterization of the p-type single crystal shows the Mg interstitials are negligible [52]. The results are reasonable considering the crystal is grown in the Mg-poor condition. Again, these results suggest that the point defects in the Mg_3_Sb_2_-based materials are highly sensitive to the chemical compositions and preparation conditions.

### 2.4. Neutron Irradiation

The challenge to identifying the electron scattering mechanism of specific defects lies in the difficulty of tuning the defects independently. To tackle this issue, we conducted the neutron irradiation experiment on the n-type Mg_3_Sb_2_-based materials. It is well known that neutron irradiation can introduce point defects (i.e., vacancies and interstitials) into the specimen but leave the grain size unaffected [64–66]. Since the sample of Mg_3.2_Sb_1.5_Bi_0.49_Te_0.01_ (prepared at 1073 K) does not show abnormal temperature-dependent electrical conductivity at room temperature, it is chosen for the neutron irradiation experiment. Detailed information for the neutron irradiation experiment can be found in Methods.

**Figure 5:**
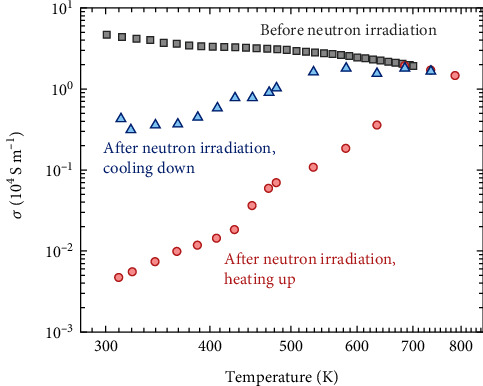
Electrical properties of Mg_3_Sb_2_-based materials prior to and after the neutron irradiation.

A comparison of the electrical properties of the sample prior to and after the neutron irradiation is shown in Figure 5. The electrical conductivity at 300 K of the pristine sample is ~4.6 × 10^4^ S m^−1^, and it reduces significantly to ~ 4.7 × 10^1^ S m^−1^ (the red symbols) after the neutron irradiation, a reduction as large as three orders of magnitude. The neutron-irradiated sample reproduces the thermal activation of electrical conductivity, which resembles that of the sample prepared at 923 K. Since the Seebeck coefficients are comparable (Figure S4, Supporting Information), the substantial difference in the electrical conductivities should mainly originate from the disparity in electron mobilities. Usually, it is the point defect that will be produced after the neutron irradiation [64, 65, 67–69]. However, due to safety concerns for radiation, we are unable to perform detailed microstructural and defect characterizations on the neutron-irradiated sample.

It should be pointed out that the electrical conductivity is partially restored after the measurement. The blue symbols represent the measurement of electrical conductivity during the cooling down period, and it is higher than that of the heating up period (the red symbols). This indicates that the concentration of the point defects reduces after the measurement, which has a similar effect as the heating treatment. In fact, the reduction of the concentration of point defects after annealing has also been reported previously [65, 67]. A similar effect has also been observed for the neutron-irradiated SiGe [70]. In addition, it should be pointed out that the as-prepared Mg_3_Sb_2_-based samples also show similar restoration of electrical conductivity during the heat and cooling cycles of the measurements [71]. Again, this indicates that the defects are highly temperature-sensitive.

### 2.5. Electron Scattering Mechanism

At this stage, however, we do not have more detailed information on the point defects, e.g., whether they are charged or not. Therefore, we cannot conclude how the point defect scatters electrons, i.e., whether it is due to the ionized impurity scattering. In case when the point defects are neutral, their effect on the electron scattering should be ascribed to the distortion of the periodic potential. However, the discussion on this topic will be further complicated by the configuration of point defects in the lattice, i.e., if point defects can be regarded as independent single point defects, or if they form complexes among themselves (e.g., defect pairs or defect clusters) [72, 73]. Therefore, identifying how the point defect scatters electrons in the n-type Mg_3_Sb_2_-based materials is a daunting task, and concerted efforts in experiments and theoretical calculations are needed to clarify this point.

We need to point out that our results do not disprove the importance of grain boundary scattering on the electrical properties of n-type Mg_3_Sb_2_-based materials [19, 20, 44]. Again, the complexity of the electron scattering mechanism of defects should be highlighted. On the one hand, there are various types of defects (e.g., grain boundaries, dislocations, and point defects) in the prepared materials, and our understanding of their specific effect on electron transport is still limited. Besides, most of the reported results are based on polycrystalline samples, which contain a high concentration of various defects. In addition, since different research groups adopt different approaches and apparatus to synthesize the samples, the type of defects and concentration of the defects can vary significantly. Then, the discussion on the defect-related phenomena in one case may not be simply applied to the others. In our case, it is more reasonable to limit the discussion on the electron scattering mechanism to the samples that have been synthesized for this work. Therefore, we cannot disapprove of the electron scattering mechanism by other defects. Discouragingly, does it mean that the electron scattering-related research cannot be reproduced and is meaningless? The answer is no. If we can conduct experiments on high-quality samples (e.g., single crystals) by intentionally introducing only one type of defect, then we should be able to clarify the electron scattering effect, and the results should be reproducible. To this end, more dedicated efforts in electron-scattering-related research are needed to advance our understanding of these topics.

## 3. Conclusion

In summary, the microstructures and defects have been investigated for the n-type Mg_3.2_Sb_1.5_Bi_0.5_, and the relationship between the electrical transport properties and point defects has been revealed. Our results show that Co-doping does not change the grain size of the Mg_3_Sb_2_-based materials, but it can still impact the electron scattering mechanism near room temperature. The synchrotron powder X-ray diffraction characterizations show that Co-doping and preparation temperature can both impact the concentration of the point defects (Mg vacancy and Mg interstitial). Combining the electrical properties and the defect characterization, it can be found that samples with lower point defect concentration exhibit higher electron mobility. In addition, neutron irradiation can significantly reduce the electrical conductivity, and it can also reproduce the thermally activated electrical conductivity that resembles that of the sample prepared at 923 K. Therefore, our results show that the point defect plays an appreciable role in the electron scattering mechanism of the n-type Mg_3_Sb_2_-based materials. Since point defects are widely present in various thermoelectric materials, their potential impact on the electron scattering mechanism deserves to be investigated.

## 4. Materials and Methods

### 4.1. Sample Preparation

Magnesium (Mg turnings), antimony (Sb shots), bismuth (Bi shots), and cobalt (Co powders) were weighted according to the composition of Mg_3.2_Sb_1.5_Bi_0.49_Te_0.01_ and Mg_3.175_Co_0.025_Sb_1.5_Bi_0.49_Te_0.01_. The weighted elements were loaded into a ball-milling jar in a glove box. Then the raw materials were ground into powders by a high-energy ball miller (SPEX 8000 M). The mechanically alloyed powder was then loaded into a graphite die under an argon atmosphere and sintered at 923 K or 1073 K under uniaxial pressure of ~50 MPa for 2 min.

### 4.2. Thermoelectric Properties Measurement

The hot-pressed pellets were cut into bar-shaped samples with dimensions of about 3 mm × 2 mm × 8 mm for simultaneous electrical resistivity and Seebeck coefficient measurement from 300 to 700 K (CTA-3, Cryoall). The Hall coefficients (*R*_H_) were measured using the van der Pauw technique under a reversible magnetic field of 1.5 T from 300 to 700 K. The Hall electron concentration (*n*_H_) was calculated using the relation *n*_H_ = 1/(*eR*_H_), and the Hall mobility (*μ*_H_) was calculated by *μ*_H_ = *R*_H_/*ρ*.

### 4.3. Microstructural Characterization

To analyze the distribution of grain size, electron back-scattering diffraction (EBSD) was performed. Square-shaped samples with a dimension of 4 mm × 4 mm × 2 mm were prepared. The samples were first ground using SiC paper and then polished by glycol-based diamond slurry and finally washed with alcohol and blown dry. After that, ion-polishing was applied to remove the surface stress. To analyze the microstructures of the samples, scanning transmission electron microscopy was performed. Selected area electron diffraction (SAED) and energy dispersive spectroscopy (STEM-EDS) were performed at 200 kV using a double Cs-corrected transmission electron microscope (JEM-ARM 200F).

### 4.4. Synchrotron Powder X-Ray Diffraction (SPXRD) Characterization

Synchrotron powder X-ray diffraction measurement was performed at the PD beamline at the Australia Synchrotron using the beamline wavelength of 0.59077 Å. All synchrotron powder X-ray diffraction samples were measured in the Debye-Scherrer geometry under transmission mode in 0.7 mm quartz capillaries sealed under an Ar atmosphere. The analyzed 2*θ* range was from 3 to 50 degrees. The Rietveld method was used to perform refinement, and the Pseudo-Voigt function was used for peak-shape fitting.

### 4.5. Neutron Irradiation

To identify the effect of Mg vacancies on the thermoelectric properties of Mg_3_Sb_2_-based materials, neutron irradiation experiments were performed. The Mg_3_Sb_2_-based materials were irradiated in the irradiation cavity of China Fast Burst Reactor-II by fast neutrons with an average energy of 1.25 MeV and fluence of 1 × 10^15^ n cm^−2^. The neutron fluence rate was about 10^9^ n cm^−2^ s^−1^ to 10^10^ n cm^−2^ s^−1^. The temperature of the sample was within the range of 300 and 343 K.

## Figures and Tables

**Table 1:** Results for the Rietveld refinement of synchrotron powder X-ray diffraction patterns.

		Mg_3.2_Sb_1.5_Bi_0.49_Te_0.01_ 923 K	Mg_3.175_Co_0.025_Sb_1.5_Bi_0.49_Te_0.01_ 923 K	Mg_3.2_Sb_1.5_Bi_0.49_Te_0.01_ 1073 K
a	Mg (1)	(0, 0, 0)	(0, 0, 0)	(0, 0, 0)
	Mg_I_	(1/3, 2/3, 0.9946)	(1/3, 2/3, 0.9493)	(1/3, 2/3, 0.8821)
	Mg (2)	(1/3, 2/3, 0.6330)	(1/3, 2/3, 0.6283)	(1/3, 2/3, 0.6345)

Occupancy	Mg (1)	0.921	0.935	0.979
	Mg_I_	0.058	0.039	0.047
	Mg (2)	1	1	1

B		0.485	0.537	0.200

R-factor	*R*_p_	2.69%	2.51%	2.57%
	*R*_wp_	3.78%	3.19%	3.71%
	*R*_exp_	1.52%	0.01%	1.39%

## Data Availability

Data associated with the current manuscript is available from the authors at reasonable request.

## Supplementary Materials

Supplementary Materials Figure S1: Carrier concentration of the prepared samples. Figure S2: Grain size information obtained by the electron backscatter diffraction characterization. Figure S3: Energy dispersive mapping characterization. Figure S4: Comparison of the Seebeck coefficient prior to and after the neutron irradiation experiment.
