# Supplementary material for: Revealing the Defect-Dominated Electron Scattering in Mg3Sb2-Based Thermoelectric Materials
Source: Research (Wash D C). 2022 Oct 14;2022:9875329. doi: 10.34133/2022/9875329 (PMC9620639; doi:10.34133/2022/9875329)
Supplement: Supplementary Materials — Figure S1: Carrier concentration of the prepared samples. Figure S2: Grain size information obtained by the electron backscatter diffraction characterization. Figure S3: Energy dispersive mapping characterization. Figure S4: Comparison of the Seebeck coefficient prior to and after the neutron irradiation experiment. [file 9875329.f1.docx]

# Supporting Information

# Revealing the defect-dominated electron scattering in Mg_3_Sb_2_-based thermoelectric materials

Jucai Jia^a^, Yan Zhou^b^, Xiaoxi Chen^c^, Wenhua Xue^a, d^, Hulei Yu^e^, Jing Li^c^, Shizhen Zhi^a^, Chen Chen^a^, Jian Wang^f^, Shuaihang Hou^f^, Xingjun Liu^a,g^, Yumei Wang^d^, Feng Cao^f^, Yue Chen^e^, Jun Mao^a, g*^, and Qian Zhang^a, g*^

*^a^ School of Materials Science and Engineering, and Institute of Materials Genome & Big Data, Harbin Institute of Technology, Shenzhen 518055, P.R. China.*

*^b^ Center for Device Thermography and Reliability (CDTR), H. H. Wills Physics Laboratory, University of Bristol, Tyndall Avenue, Bristol BS8 1TL, UK.*

*^c^ Institute of Nuclear Physics and Chemistry, China Academy of Engineering Physics, Mianyang, 621900, China.*

*^d^ Beijing National Laboratory for Condensed Matter Physics, Institute of Physics, Chinese Academy of Sciences, 100190 Beijing, P.R. China.*

*^e^ Department of Mechanical Engineering, The University of Hong Kong, Pokfulam Road, Hong Kong SAR, China.*

*^f^ School of Science, Harbin Institute of Technology, Shenzhen 518055, P.R. China.*

*^g^ State Key Laboratory of Advanced Welding and Joining, Harbin Institute of Technology, Harbin 150001, P.R. China.*

^*^Corresponding authors, email: [maojun@hit.edu.cn](mailto:maojun@hit.edu.cn); [zhangqf@hit.edu.cn](mailto:zhangqf@hit.edu.cn).

## Content

**1. Carrier concentration of the prepared samples;**

**2. Grain size information obtained by the electron backscatter diffraction characterization;**

**3. Energy dispersive mapping characterization;**

**4. Comparison of the Seebeck coefficient prior to and after the neutron irradiation experiment.**

## 1. Carrier concentration

**Fig. S1**. Hall carrier concentration of Mg_3.2_Sb_1.5_Bi_0.49_Te_0.01_ (hot pressed at 923 K and 1073 K) and Mg_3.175_Co_0.025_Sb_1.5_Bi_0.49_Te_0.01_ (hot pressed at 923 K).

## 2. Grain size information


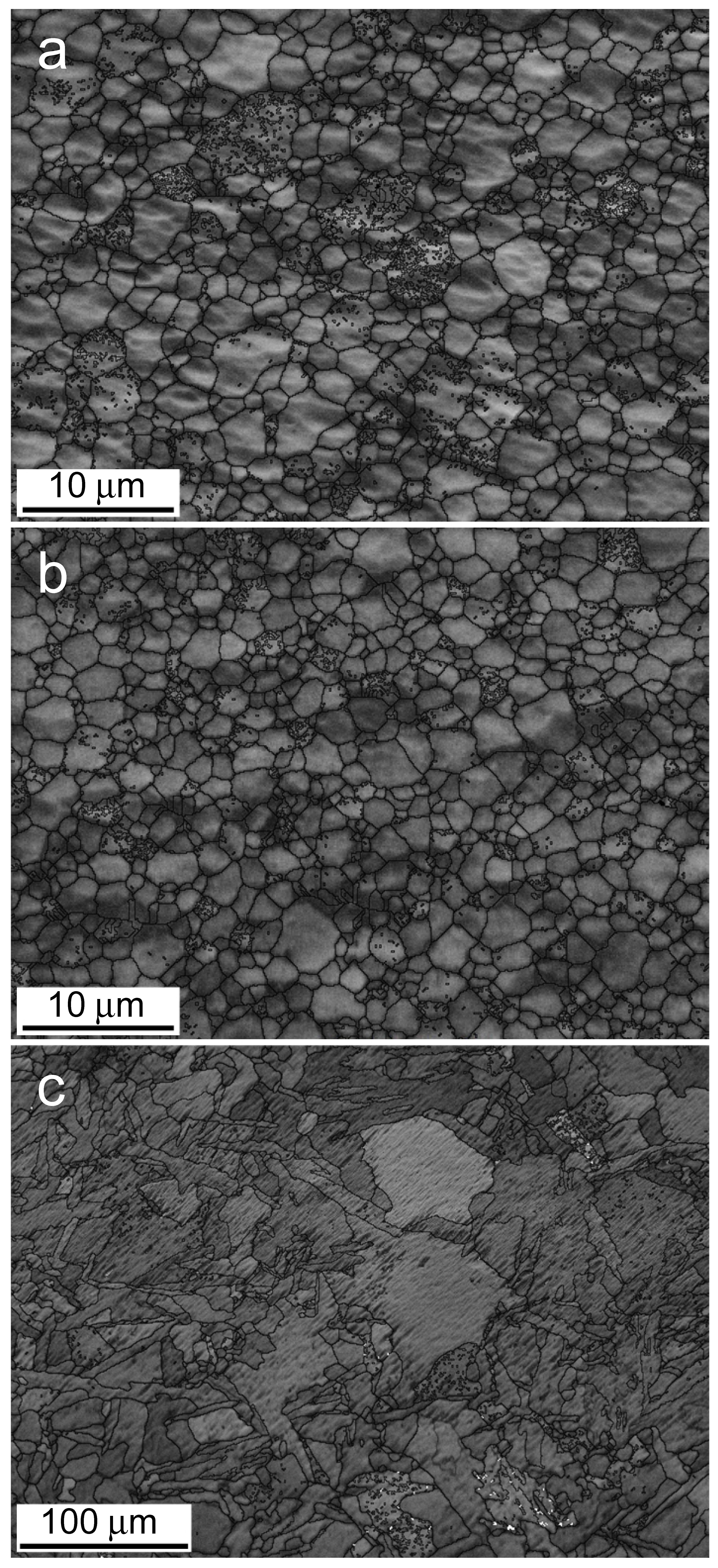


**Fig. S2. EBSD images**. (a)(c) Mg_3.2_Sb_1.5_Bi_0.49_Te_0.01_ that is hot-pressed at 923 K and 1073 K, respectively, and (b) Mg_3.175_Co_0.025_Sb_1.5_Bi_0.49_Te_0.01_ that is hot-pressed at 923 K.

## 3. Energy dispersive spectroscopy


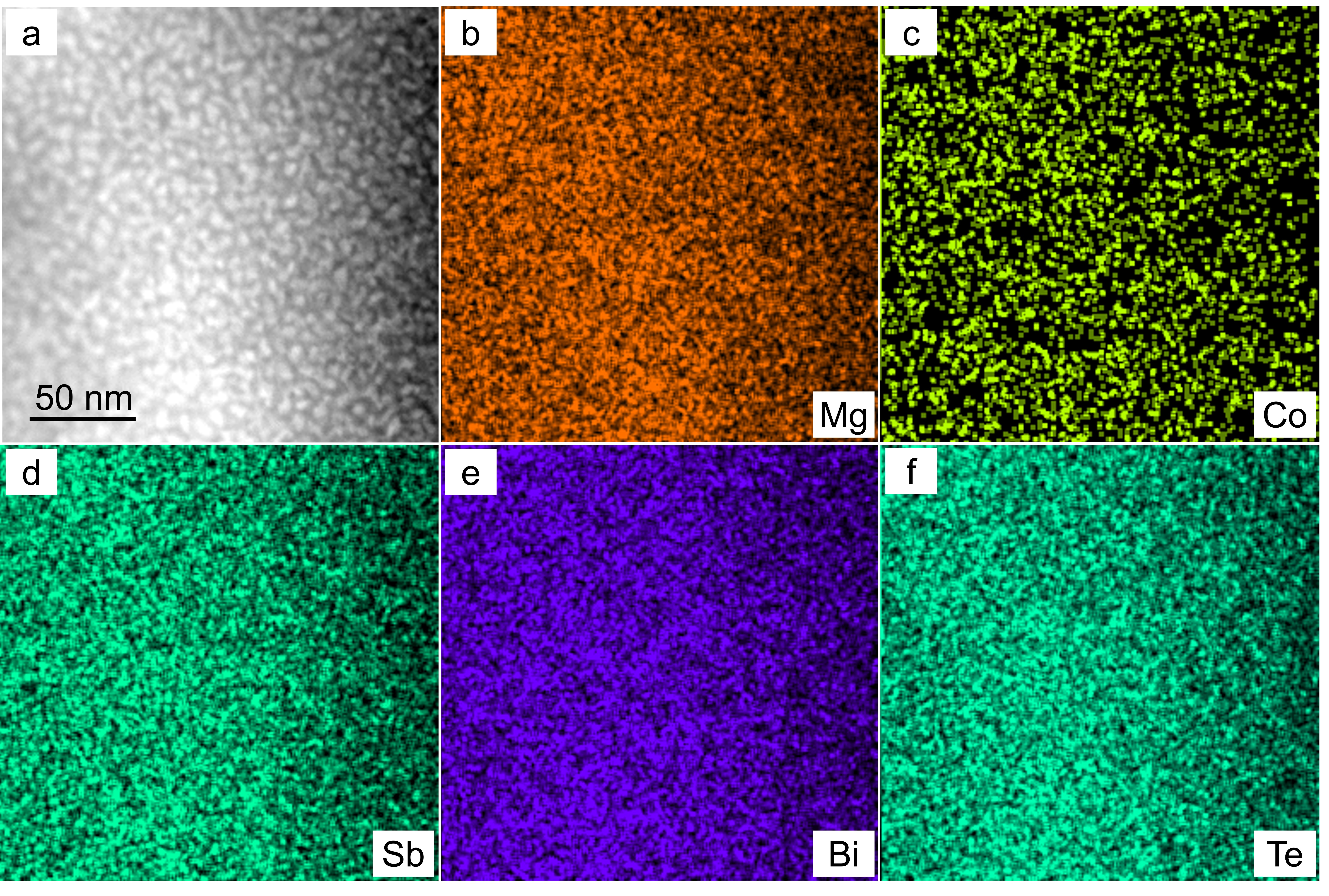


**Fig. S3.** EDS mapping of the Mg_3.175_Co_0.025_Sb_1.5_Bi_0.49_Te_0.01_.

## 4. Seebeck coefficient after neutron irradiation

**Fig. S4.** Comparison of the Seebeck coefficient prior to and after the neutron irradiation experiment.
